# Construction of Recombinant Bacmid Containing M2e-Ctxb and Producing the Fusion Protein in Insect Cell Lines

**DOI:** 10.5812/ircmj.13176

**Published:** 2014-02-07

**Authors:** Nima Mirzaei, Talat Mokhtari Azad, Rakhshandeh Nategh, Hoorieh Soleimanjahi, Nour Amirmozafari

**Affiliations:** 1 Department of Biology, Science and Research branch, Islamic Azad University, Tehran, IR Iran; 2 Department of Virology, Tehran University of Medical Sciences, Tehran, IR Iran; 3 Department of Virology, Tarbiat Modares University, Tehran, IR Iran; 4 Department of Microbiology, Tehran University of Medical Sciences, Tehran, IR Iran

**Keywords:** Influenza Vaccines, Baculovirus, Cholera Toxin Subunit B

## Abstract

**Background:**

Sequence variations in glycoproteins of influenza virus surface impel us to design new candidate vaccines yearly. Ectodomain of influenza M2 protein is a surface and highly conserved protein. M2e in influenza vaccines may eliminate the need for changing vaccine formulation every year.

**Objectives:**

In this study, a recombinant baculovirus containing M2e and cholera toxin subunit B fusion gene was generated with transposition process to express in large amounts in insect cell lines.

**Materials and Methods:**

M2e-ctxB fusion gene was created and cloned into pFastBac HT. The recombinant vector was transformed into DH10Bac cells to introduce the fusion gene into the bacmid DNA via a site-specific transposition process. The recombinant bacmid was then extracted from white colonies and further analyzed using PCR, DNA sequence analyzing, and indirect immunofluorescence assay.

**Results:**

PCR and DNA sequence analyzing results showed that the fusion gene was constructed as a single open reading frame and was successfully inserted into bacmid DNA. Moreover, indirect immunofluorescence results showed that the fusion gene was successfully expressed.

**Conclusions:**

Baculovirus expression vector system is valuable to produce M2e based influenza vaccines due to its simple utilization and ease of target gene manipulation. The expressed protein in such systems can improve the evaluating process of new vaccination strategies.

## 1. Background

Influenza is one of the most serious public health predicaments in the world (1-4) Influenza virus A has a potential ability to cause epidemic and pandemic flu via two relatively close manners. Surface antigenic variations, commonly named as antigenic drift, and reassortment of RNA segments among different circulating strains of the virus commonly named as antigenic shift (5, 6) are the two mechanisms. These variations usually occur yearly due to the circulation of the virus among the population allowing the virus to escape host acquired and innate immune responses. Such abilities coerce us to design and change influenza vaccine formulations every year (2, 7). Many investigations have been performed to design and produce more effective vaccines to prevent spreading pandemic influenza (8-10). An effective influenza vaccine should be developed based on the genetically stable antigens (11). One of the most conserved influenza antigens is external-domain of M2, briefly named as M2e. M2 helps to translocate H+ ions to endocytosed virions and acts as a modulator of pH (12-14). Despite the genetic stability, M2e weak immunogenicity is the most important concern for using it as an influenza vaccine. It usually fails to illicit an effective immune response against the virus (6).

Using adjuvants and genetic tags to other potent immunogens such as heat shock protein 70 is suggested to enhance M2e immunogenicity (15). Cholera toxin subunit B (CtxB) would be an applicable antigen to develop fusion vaccines due to its potential ability to induce mucosal immune responses (16). For evaluating efficiency and immunogenicity of fusion vaccines, a large amount of recombinant antigen should be provided. Different types of expression systems have been developed to produce recombinant proteins. Prokaryotic and eukaryotic expression systems are the two major well-characterized expression systems widely used to produce recombinant proteins. Prokaryotic expression systems apply bacterial cells as host and mainly used for production of rather uncomplex protein and peptides. Eukaryotic expression systems have been studied in the last decades, mainly based on yeast, mammalian or insect cells to produce complex recombinant proteins. Although prokaryotic systems can be useful for protein production, these systems have some limitations. For example, they cannot glycosylate the expressed proteins and therefore some of eukaryotic proteins expressed in *E. coli* and other prokaryotic systems are non-functional. Moreover, the bacterial expressed proteins are usually mixed with endotoxin derived from the bacterial cell wall. These limitations have many implications in downstream process and studies such as vaccine research (17). It has become a common expression system for both basic research and large-scale commercial applications. A key factor to the popularity of insect cell expression is the ability of insect cells to produce relatively large quantities of post-translationally modified eukaryotic proteins in a relatively short period. Most insect cell-produced proteins have been expressed by employing the Baculovirus Expression Vector System (BEVS). BEVS is suitable to produce large amounts of recombinant protein in insect cell lines such as Sf9 and Sf21. The BEVS is an approved system for heterologous expression and production of viral antigens with vaccine potential for humans and animals. BEVS has been widely used for production of subunit vaccines against parasitic diseases as well. BEVS uses transport system present in higher eukaryotic cells providing conditions more similar to the human cells. Moreover, there are a wide variety of vectors and culture media adapted for this system making it simple to use (18, 19).

Different vectors have been developed for expressing the target gene in insect cell lines. Most BEVSs use Autographa californica Multiple Nuclear Polyhedrosis Virus (AcMNPV) as the prototype baculovirus. Due to the large size of the AcMNPV genome (134 Kb), a homologous recombination or transposition is required to insert the target gene into the baculovirus genome. In practice, the target gene is subcloned into a transfer vector containing a suitable promoter flanked by a part of baculovirus DNA derived from non-essential locus such as the polyhedrin gene of AcMNPV to perform recombination with the baculovirus genome. The recombinant viral DNA is then transfected into the insect cell lines. To do this process easily, the baculovirus genome has been modified, so that it can be maintained in E.coli. This form of baculovirus DNA which can be maintained and amplified in both E.coli and insect cell is named bacmid. Moreover, recently manipulated baculovirus genome (bacmid) has other features such as the ability to perform site-specific transposition for faster inserting the foreign gene, by adding Tn7, and ease of selection, by introducing lacZ cassette into the bacmid DNA. The bacmids are usually carried by special strains of *E. coli* such as DH10Bac (20). Appearing such modified BEVSs help us to evaluate new generation of vaccines more effectively in comparison with other expression systems.

## 2. Objectives

The aim of this study was to produce recombinant M2e-ctxB fusion protein using Baculovirus Expression Vector System via a site-specific transposition able to insert the fusion gene into bacmid DNA and evaluating expression of the fusion protein in insect cell line by indirect immunofluorescence assay.

## 3. Materials and Methods

### 3.1. Bacterial Strain and Viruses

Toxigenic Vibrio cholera strain 569B was employed to amplify ctxB. Influenza A/Puerto Rico/8/34 was used as standard strain to amplify M2e. E.coli strain Top10 (F- end A1 recA1) (Invitrogen, USA) was used for transformation and amplifying the recombinant vector pFastBac HT/M2e-ctxB. E.coli Top10 is an appropriate strain to perform the cloning process. Construction of the recombinant baculovirus genome containing M2e-ctxB (recombinant bacmid) was performed in E.coli strain DH10Bac (F- endA1 lacZ∆M15) (Invitrogen, USA).

### 3.2. Plasmid and Cloning Vectors

To generate recombinant bacmid pFastBac HT was used as the transfer vector. E. coli strain DH10Bac contained the baculovirus modified DNA (bacmid) with a mini-attTn7 target site and the helper plasmid. The helper plasmid harbored by DH10Bac strains, confers resistance to tetracycline and encodes enzymes needed for transposition of the gene of interest onto the bacmid. Therefore, adding appropriate concentration of tetracycline into the agar plates is very important to maintain helper plasmid in *E. coli* DH10Bac. The transfer vector carried mini-Tn7 element, polyhedron promoter and N-terminal 6X histidine tag.

### 3.3. PCR Amplification

M2e was amplified using influenza virus A/Puerto Rico/8/34 genome as the template with the following primers:

F: 5'-CGGGATCCACCATGTCCCTGCTGACCGAGG-3'

R: 5'-CCCAAGCTTAGCCATCGCTGCTGCCATT-3'

The forward primer contained BamHI restriction site (in bold) and start codon (the underlined nucleotides). The Reverse primer contained HindIII restriction site, shown in bold type. Oligo software V. 7 was used to design primers. The RT-PCR and PCR reaction was performed using super script III one step RT-PCR kit (Invitrogen, USA) in a single tube containing 25 µL reaction mix, 1 unit RT/Taq DNA polymerase, Template (1 µg), 1 µL of F and R primers (10 pmol for each), and water nuclease-free to 50 µL final volume. The PCR program included the following steps: cDNA synthesis at 48ºC for 30 min, denaturation at 94ºC for 2 min, followed by 40 cycles of 94ºC for 15 sec (denaturation), 58ºC for 30 sec (annealing), 68ºC for 1 min (extention), and a final extension at 68ºC for 5 min. Genomic DNA from Vibrio cholera 569B was isolated using Gene Jet genomic DNA purification kit (Thermo scientific, USA). The PCR reaction was performed using the primers F: 5'-CCCAAGCTTATTAAATTAAAATTTGGTG-3'

R: 5'-CGGAATTCTTAATTTGCCATACTAATTG-3'

HindIII and EcoRI restriction sites (in bold) were added into the F and R primers, respectively. Oligo software V. 7 was used to design primers. PCR reaction was performed in a tube containing 5 µL of 10x PCR buffer, 1 µL of dNTP mix (0.2 mM for each), 1.5 µL of MgCl2 (1.5 mM), 1 µL of each primer (10 pmol for each), 1-2 µL of template DNA, 1 unit of platinum Taq DNA polymerase (Invitrogen, USA) and water nuclease-free up to 50 µL final volume. The PCR program included the following steps: denaturation at 94ºC for 1 min, followed by 30 cycles of 94ºC for 30 sec (denaturation), 62ºC for 30 sec (annealing), 72ºC for 1 min (extension), and a final extension at 72ºC for 5 min. All the PCR products were then purified using PCR purification kits (Bioneer, South Korea) and sequenced.

### 3.4. Fusion of M2e to ctxB

The amplified M2e and ctxB were gel purified and digested with HindIII (Thermo scientific, USA) separately. The digested products were ligated using T4 DNA ligase (Invitrogen, USA) and incubated overnight at room temperature to create M2e-ctxB fusion gene. A PCR was performed directly on ligation product using M2e forward and ctxB reverse primers to amplify the fusion fragments. The amplified M2e-ctxB fragments were purified and sequenced using the BigDye® Terminator v3.1 Cycle Sequencing Kit (Invitrogen, USA) to confirm the accuracy of the fusion process.

### 3.5. Cloning and Construction of pFastBac HT/M2e-ctxB

The PCR product was gel purified and double digested with BamHI and EcoRI (Thermo scientific, USA) and then ligated into BamHI and EcoRI pre-digested pFastBac HT (Invitrogen, USA). The ligation product was incubated overnight at room temperature and then used for transformation of *E. coli* strain Top10. The transformants were plated on LB agar plates containing 100µg/mL ampicillin and incubated at 37 ºC for 24 h.

### 3.6. Generating Recombinant Bacmid Containing M2e-ctxB

After cloning, E.coli strain DH10Bac cells which carried the bacmid DNA, as well as kanamycin and tetracycline resistance genes, were chemically transformed with 1ng of the purified pFastBac HT/M2e-ctxB. The recombinant bacmid was created by culturing the transformants in LB broth. Following incubation at 37 ºC in a shaker (225 rpm) for 4h to allow expression of the antibiotic resistance genes, insertion of the mini-Tn7 containing M2e-ctxB from pFastBac HT into the mini-attTn7 attachment site on bacmid DNA was performed. After the incubation period serial dilutions (10^−1^, 10^−2^, 10^−3^) of the cells were prepared with LB broth and 100 µL of each dilution was plated on LB agar containing 7 µg/mL gentamicin, 50 µg/mL kanamycin, 10 µg/mL tetracycline, 40 µg/mL IPTG, and 100 µg/mL X-gal (Invitrogen, USA). The plates were incubated at 37 ºC for at least 48h until the blue and white colonies were appeared. The white colonies were picked and restreaked on LB agar containing the above mentioned concentration of antibiotics, IPTG and X-gal.

### 3.7. PCR Analyzing of the Recombinant Bacmid

To verify successful insertion of the M2e-ctxB fusion gene into the bacmid DNA, the purified recombinant bacmid was analyzed by PCR. A single colony with a white phenotype was picked and inoculated into LB broth containing appropriate concentrations of kanamycin, gentamicin and tetracycline. Following incubation at 37 ºC at 225 rpm for 18-24 h, the cells were harvested and recombinant bacmid was purified manually according to the protocol provided by the manufacturer (Invitrogen, USA). Presence of the M2e-ctxB in bacmid DNA was verified by PCR using a combination of pUC/M13, gene specific primers and each set of primers lonely. pUC/M13 primers were custom synthesized with the following sequences: 

pUC/M13 F: 5'-CCCAGTCACGACGTTGTAAAACG-3'

pUC/M13 R: 5'-AGCGGATAACAATTTCACACAGG-3'

PCR was performed according to the manufacturer’s instructions and analyzed by agarose gel electrophoresis.

### 3.8. Transfection and Infection of Sf9 Cells by Recombinant Bacmid

The precultured Sf9 cells were diluted by serum free medium to 1×10^6^ cells /mL and then transfected by recombinant bacmid using Cellfectin II reagent (Invitrogen, USA). The recombinant bacmid was collected after 72h according to the manufacturer instruction. To produce M2e-CtxB fusion protein, the Sf9 cells were seeded at the density of 1−2 × 10^7^ per flask (75 cm^2^) and infected by recombinant bacmid collected from the last stage at MOI (Multiplicity of Infection) 3 in 10 mL of Sf-900 III SFM (Invitrogen, USA). All the flasks were incubated at 27 °C.

### 3.9. Indirect Immunofluorescence Assay

To confirm expression of M2e-CtxB fusion protein, indirect immunofluorescence assay was performed using anti-beta subunit cholera toxin FITC conjugated antibody (Abcam, UK). At 6h post-infection Sf 9 cells were washed with PBS and fixed with cold acetone. Production of M2e-CtxB fusion protein was detected by the anti-cholera toxin B subunit monoclonal antibody diluted in PBS. The antibodies were removed and cells were washed three times with PBS and then incubated with the secondary at 37 °C for 20 min. The cells were subsequently re-washed with PBS and visualized by immunofluorescence microspore.

## 4. Results

### 4.1. PCR Amplifying of the M2e and ctxB

The fragments M2e and ctxB were successfully amplified by PCR. The amplified M2e fragment was about 80 bp and ctxB amplified fragment was 380 bp. The amplified fragments were then fused to construct M2e-ctxB amplified by PCR. M2e-ctxB fusion fragment was about 460 bp. Sequence data showed that M2e was successfully attached to the 5' end of ctxB and a single open reading frame was generated.

### 4.2. Cloning and Construction of pFastBac HT/M2e-ctxB

**Figure 1.**
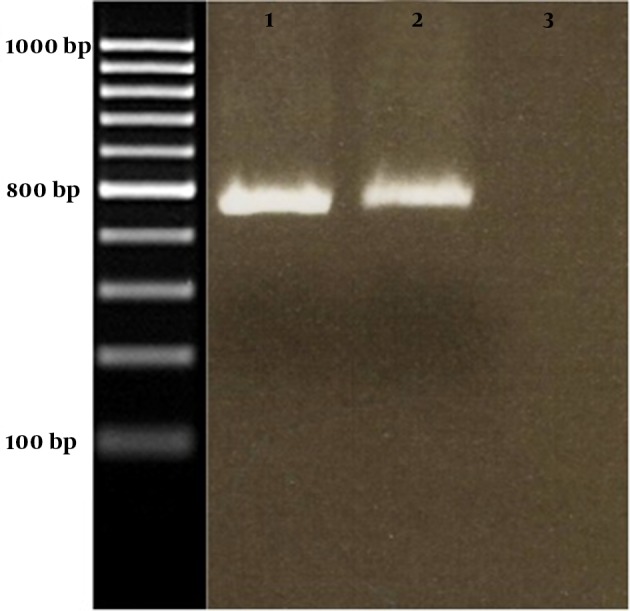
Gel Electrophoresis of PCR Products Line 1, 2: M2e-ctxB, 460 bp in size; Line3: negative control

The amplified fusion fragments were then cloned into pfastBac HT to form pFastBac HT/M2e-ctxB. E.coli strains Top10 was transformed by pFastBac HT/M2e-ctxB and colony PCR was performed to confirm insertion of the M2e-ctxB fusion gene into pFastBac HT. All the selected colonies carried the recombinant pFastBac HT/M2e-ctxB based on the results of agarose gel electrophoresis. The amplified fragment was approximately 460 bp in all the selected colonies (Figure 1). The sequence of fusion gene and accuracy of the cloning process were confirmed by DNA sequence analyzing. DNA Sequence analyzing showed that M2e was successfully placed in the 5' end of ctxB.

### 4.3. Construction of Recombinant Bacmid

The purified recombinant pFastBac HT/M2e-ctxB was transformed into E.coli strain DH10Bac to generate recombinant bacmid containing M2e-ctxB. After 48h incubation, the white and blue colonies appeared on plates containing kanamycin, tetracycline, gentamicin, X-gal and IPTG. Some white colonies were selected and restreaked on agar palates to ensure whether they show the white phenotype.

### 4.4. PCR Analysis of Recombinant Bacmid

**Figure 2.**
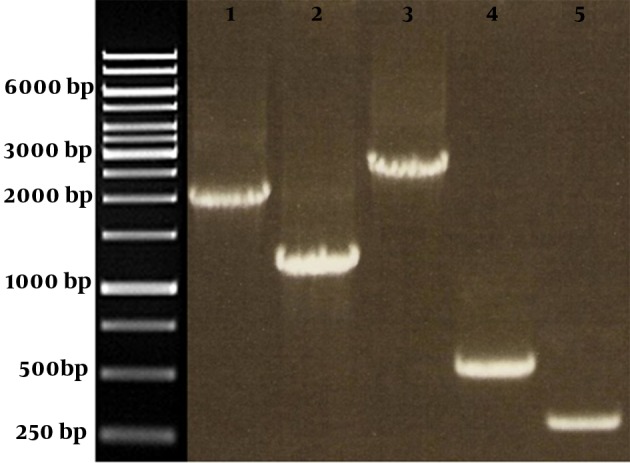
Pattern of PCR Reaction on Recombinant Bacmid Using a Combination of Different Primers Lane1: PCR reaction using pUC/M13 F and M2e-ctxB R; Lane2: PCR reaction using M2e-ctxB F and pUC/M13 R; Lane 3: PCR reaction using pUC/M13 F and R; Lane4: PCR reaction using M2e-ctxB F and R; Lane5: PCR reaction using pUC/M13 F and R and non-recombinant bacmid as template.

We performed a series of PCR reactions for final confirmation of the recombinant bacmid construction. The PCR reactions were performed using a combination of backbone and gene specific primers to avoid any false results due to contamination. The amplified fragments using pUC/M13 F and M2e/ctxB R, M2e/ctxB F and pUC/M13 R, pUC/M13, and M2e/ctxB forward and reverse primers were between 460 and 2900 bp in size. PCR reaction using non-recombinant bacmid DNA as template amplified pUC/M13 priming region. The corresponding bond on agarose gel was almost 300 bp in size (Figure 2). All primer sequences and the amplified fragments were shown in Table 1. 

**Table 1.** Details of Amplified Region and Primer Sets Used for PCR Analysis of Recombinant Bacmid

Primer Pairs	Sequence (5' to 3')	Fragment ^a^Size (bp)	The Amplified Region
**pUC/M13 F M2e/ctxB R**	CCCAGTCACGACGTTGTAAAACG CGGAATTCTTAATTTGCCATACTAATTG	2200	Tn7 R + Polyhydrin promoter + M2e/ctxB fusion gene
**M2e/ctxB F pUC/M13 R**	CGGGATCCACCATGTCCCTGCTGACCGAGG AGCGGATAACAATTTCACACAGG	1200	M2e/ctxB fusion gene + Tn7 L
**pUC/M13 F pUC/M13 R**	CCCAGTCACGACGTTGTAAAACG AGCGGATAACAATTTCACACAGG	2900	Tn7 R + Polyhydrin promoter + M2e/ctxB fusion gene + Tn7 L
**M2e/ctxB F M2e/ctxB R**	CGGGATCCACCATGTCCCTGCTGACCGAGG CGGAATTCTTAATTTGCCATACTAATTG	460	M2e/ctxB fusion gene

^a^ All the Fragment Sizes are Expressed Approximately and Has Been Calculated According to the Sequence date of the Bacmid DNA

### 4.5. Indirect Immunofluorescence Assay

**Figure 3.**
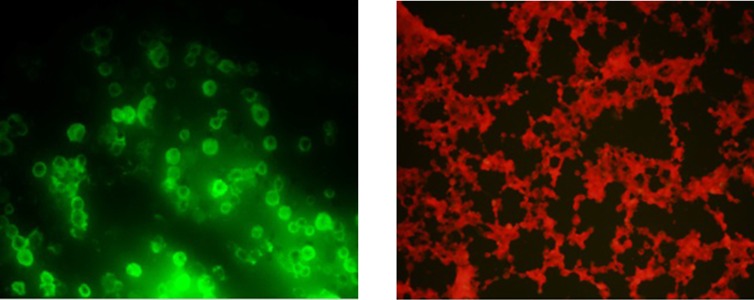
Verification of Recombinant Bacmid Construction Containing M2e-ctxB Fusion Gene Using Indirect Immunofluorescence Sf9 Cells Expressing M2e-ctxB (A), Sf9 Cells Containing non-Recombinant Bacmid DNA as Negative Control (B)

The infected Sf9 cells were analyzed by fluorescence microscopy to verify production of the recombinant protein. We successfully detected green fluorescence in Sf9 cell infected with the recombinant bacmid. We also used the non-infected Sf9 cells as negative control to compare with the infected cells to evaluate green fluorescence emitting (Figure 3). 

## 5. Discussion

Most influenza vaccines are based on influenza surface glycoproteins hemagglutinin (HA) and neuraminidase (NA). These antigens are subjected to antigenic drift and antigenic shift resulting in epidemic and pandemic influenza around the world. The most recent pandemic occurred in 2009 due to H1N1 influenza A virus. All continents were affected by this new strain, which had enormous economic consequences. The mortality rate varied in different countries; for example in Iran, of the 3672 confirmed cases of influenza, 140 deaths were reported (21, 22). Genetic stability is the most important reason for increasing interest on matrix 2 ectodomain domain (M2e) in developing new flu vaccines (23, 24). M2e cannot potentially stimulate human immune system; however, immunological responses may increase when we use it along with a potent immunogen. In most of these cases, M2e is genetically fused to some other proteins. The proteins could be a single antigen such as rotavirus NSP4 (25) or a composition of different particles. Hashemi et al. fused M2e gene to the gene encoded capsid protein of T7 bacteriophage and expressed them in E. coli. M2e-T7 fusion particle enhanced the immunogenicity of mice against influenza virus successfully (26). Cholera toxin subunit B (CtxB) in the form of fusion gene can effectively enhance the immune responses against the fused gene (27, 28). The sequencing results showed that M2e was successfully attached to ctxB and the M2e-ctxB fusion fragment was constructed as a single open reading frame. Razavi et al. constructed HSP70-E7 fusion gene as a tool in HBV vaccine research (15). Ebrahimi et al. generated a fusion gene containing M2e and HSP70 and cloned into pPICMH cloning vector as a same manner we did. They also used a serine and glycine codon to separate the two adjacent genes (29).

Different systems, including prokaryotic and eukaryotic expression methods, have been used for production of recombinant viral proteins. Hence, choosing a proper and appropriate expression system is very important to produce viral efficient vaccines. Kim et al. used the prokaryotic expression system to produce M2e. They expressed M2e in Escherichia coli and used it along with an inactivated H9N2 virus vaccine and evaluated the mice immune response against the influenza virus (30). Different eukaryotic expression systems including yeast and mammalian systems have also been frequently used for production of M2e fusion genes (28, 31) Baculovirus expression vector system (BEVS) is one of the most powerful systems to produce recombinant proteins. Use of BEVSs was increased noticeably after production of the first protein (human Beta interferon) in insect cells infected with baculovirus (32). Using BEVS takes advantages of high level production of biologically active and properly folded functional recombinant protein. However, there are some limitations for using BEVS as an expression system due to difficulty of transferring the recombinant baculovirus into the insect cells (33-35). Incomplete influenza viruses are recently taken into the consideration. These defective viruses usually consist of three or more virus structural proteins such as HA, NA and M, which are self-assembled to construct non-infectious particles called virus like particle (VLPs). VLPs are used in vaccine development and most commonly produced by BEVS (36). Rezaei et al. constructed a recombinant bacmid containing HA gene and expressed in Sf9 cell line. The expressed protein was successfully assembled to generate influenza VLPs (37). Safdar et al. used BEVS to produce M2 for safe production of influenza vaccine. They showed that the insect cell-baculovirus production technology is a modern solution for rapid influenza antigen production, and that this technology is particularly suitable for influenza where annual adjustment of the vaccine is required (38). The same results obtained in a study performed by Slepushkin et al. They induced protective immunity in Balb/c mice against influenza A/Ann Arbor/6/60 virus using baculovirus-expressed M2e (39). Once the transfer plasmid (pFastBac HT) is transformed into *E. coli* DH10Bac, transposition is mediated by the enzymes encoded by the helper plasmid between the mini-Tn7 element on the pFastBac HT and the mini-attTn7 target site on the bacmid to generate a recombinant bacmid. Bacmid is a shuttle vector replicated in both *E. coli* and insect cell lines such as SF9 and SF21.

The white and blue colonies appeared on agar plates containing antibiotics, X-gal and IPTG. The appearance of white colonies showed the success of recombinant bacmid construction in DH10Bac cells. Insertion of M2e-ctxB into the mini-attTn7 site of bacmid DNA disrupted the sequence and subsequently prevented expression of LacZα resulted in appearance of white colonies in the presence of X-gal and IPTG. In contrast, blue colonies on agar plates represent the DH10Bac cells harboring unaltered (non-recombinant) bacmid. Bacmid DNA is normally greater than 135000 bp in size (19). Since restriction analysis of a DNA fragment with this size is difficult to perform. Moreover, this method is not efficient to verify successful transposition of the fused gene into the bacmid. Agarose gel analyzing is recommended to confirm the bacmid DNA extraction. However, due to some limiting factors such as high molecular weight, long electrophoresis and presence of different DNA bands on agarose gel, it is not recommended to verify the insertion of the target gene onto the bacmid.

A PCR reaction using a combination of pUC/M13 and gene specific primers is the most convenient technique to analyze the recombinant bacmid. Mini-att T7 site, the insertion site of target gene, on bacmid DNA is flanked by pUC/M13 priming regions verifying the presence of the target gene. Gel electrophoresis analysis showed that the PCR reaction amplified the region containing M2e-ctxB on bacmid DNA using different combinations of the primers. Such results did not obtain for the non-recombinant bacmid DNA. These results demonstrated that the M2e-ctxB fusion fragment was successfully inserted into the bacmid DNA. The same results achieved by Salmani et al. where the different sizes of PCR products showed that influenza virus M1 gene transposed into the bacmid DNA (40). The final verification of recombinant bacmid construction was performed by indirect immunofluorescence assay using mAb. All the infected Sf9 cells emitted green fluorescence indicating the presence of the recombinant protein containing M2e-ctxB. This assay indirectly showed that the recombinant bacmid was successfully constructed. In this study, a recombinant bacmid was generated using two simple steps in which M2e-ctxB was inserted into Mini-att T7 site of the bacmid DNA. Indeed the first phase, production of M2e-ctxB fusion protein, was successfully performed. We suggest using BEVS to produce fusion proteins due to its simple use and working flexibility. Moreover, using BEVS can produce a large amount of recombinant protein compared to the other expression systems, which is highly recommended for evaluation of new vaccines.
